# Access, timeliness and retention for HIV testing under early infant diagnosis (EID) program, India

**DOI:** 10.1038/s41598-023-32056-y

**Published:** 2023-04-06

**Authors:** Suchit Kamble, Nilesh Gawde, Noopur Goel, Mohan Thorwat, Kalyani Nikhare, Shilpa Bembalkar, Sushmita Kamble, Radhika Brahme, Swapna Pawar, Rakesh Sahoo, Manish Rana, Manishkumar Singh, Syed Ahmed Mohiuddin, Shivappa Hatnoor, Bayapa Reddy Narapureddy, M. Saleem, Kirti Shekhawat, Vinita Verma, Neha Kapoor, Chinmoyee Das, Raman Gangakhedkar

**Affiliations:** 1 ICMR - National AIDS Research Institute, Pune, Maharashtra India; 2 Tata Institute of Social Sciences, Mumbai, Maharashtra India; 3 GMERS Medical College, Sola, Ahmedabad, Gujarat India; 4 Dr. Ram Manohar Lohiya Institute of Medical Sciences, Lucknow, Uttar Pradesh India; 5 Osmania Medical College, Hyderabad, Telangana India; 6 RIMS, Raichur, Karnataka India; 7 College of Applied Medical Science, King Khalid University, Abha, Kingdom of Saudi Arabia; 8 Institute of Community Medicine, Madurai Medical College, Madurai, Tamil Nadu India; 9 SP Medical College, Bikaner, Rajasthan India; 10 National AIDS Control Organisation, New Delhi, India

**Keywords:** Diseases, Health care

## Abstract

Early Infant Diagnosis of HIV infection services are crucial for managing the perinatally acquired HIV infection. Assessing the performance of the EID services and its underlying determinants is important for the National AIDS Control Program, India. The objectives of this study were to find out access to HIV testing, the timeliness of the testing cascade, and the proportion of HIV exposed infants who are followed up to 18 months for a definitive diagnosis of HIV. The study design was a mixed method. A total of 11 states accounting for 80% of HIV-positive pregnant women were selected. Program records from a total of 62 Integrated counselling and testing centres (ICTCs) served as the source of information. The qualitative component included interviews of program managers at the state and district level, service providers at the ICTC level, and caregivers of HIV exposed infants. In the sampled 62 ICTCs, 78% of the HIV exposed infants had at least one HIV test. Of the infants who had HIV tests, 50% had at first sample collected by 8 weeks of age. The median turnaround time from sample collection to DNA PCR testing was 36 (IQR 19–70) days and that to next sample collection in case of detection of virus in the first sample was 66 (IQR 55–116) days. At 18 months of age, 544 (62%) HIV exposed infants were retained in the EID testing cascade. A total of 30 infants were diagnosed with HIV at a median age of 421 (IQR 149–650) days. More than three fourth of the HIV exposed infants had access to early infant diagnosis (EID) services. Both demand and supply-side factors contribute to access, timeliness and retention and there is a need to address these factors.

## Introduction

Early infant diagnosis (EID) of HIV infection and subsequent initiation of anti-retroviral therapy (ART) significantly helps in the reduction of HIV related morbidity and mortality among children born to HIV positive mothers. The children with HIV early anti-retroviral therapy (CHER) trial provided much-needed empirical evidence that early therapy against perinatal HIV infection can help reduce early infant mortality^1^. It was imperative that HIV should be diagnosed early enough to enable initiation of anti-retroviral therapy (ART) in time. India like most other low-middle income countries was providing diagnostic services with help of rapid antibody tests through its National AIDS Control Program (NACP). While these rapid antibody tests have the advantage of detecting antibodies to HIV in a short time and could be administered in peripheral health facilities, they face a distinct disadvantage of not being able to differentiate maternally acquired HIV antibodies from the ones developed by infants due to perinatally acquired infection. The CHER trial evidence led to adopting nucleic acid test for early infant diagnosis of HIV infection. The National AIDS Control Program (NACP) of India launched the early infant diagnosis (EID) services in 2010 and aimed to scale it up to all the integrated counselling and testing centres (ICTCs) during phase IV of NACP (2012–2017).

India is committed to the elimination of mother to child transmission of HIV and aims for universal access to HIV testing to all pregnant mothers. Every year, about 22,000 pregnant mothers are estimated to be HIV infected and children born to them need access to EID services. This number is spread over wide geography of the country with very low numbers at sub-district and facility levels. In 2019–2020, about 15,000 were diagnosed at one of the 5500 stand-alone Integrated Counselling and Testing Centres (SA-ICTC)^2^. Select SA-ICTCs were prioritised by NACP to also serve as EID sample collection centres in the form of dried blood spots (DBS) and are referred to as EID-ICTCs in this paper. SA-ICTCs without a facility for EID sample collection are linked to EID-ICTCs so that all HIV positive pregnant women can be referred to EID-ICTCs for availing HIV testing of their infants.

Under India’s EID services, the diagnostic algorithm warrants detection of HIV-1 virus in two samples with deoxyribonucleic acid—polymerase chain reaction (DNA PCR) test. The second sample is collected only if the first one detects HIV-1. At the EID ICTCs, DBS is collected from HIV exposed infant (HEI) on filter paper by heel/finger/toe prick and sent to a specialized Regional Reference Laboratory (RRL), where the DNA PCR test is performed. Under the EID program, NACO has established a network of seven regional reference laboratories (RRL). As per guidelines, samples are to be collected at 6 weeks, 6 months and 12 months of age and six weeks after the stoppage of breastfeeding. All HEIs are expected to undergo an antibody-based HIV test at 18 months of age for confirmation of diagnosis. Literature from low-middle income countries (LMICs) especially from sub-Saharan Africa underscore challenges to implementation of HIV EID services especially high attrition from EID services^3^.

There is limited literature from India on the reach, effectiveness and challenges of the EID services. The studies include those limited to a centre^4,5^ or a state and were conducted shortly after the launch of EID^6–8^. Findings from these studies indicated delays along the EID testing cascade in those specific settings in the initial years of the national programme. But these the programme has been addressing over the years. There is also a lack of nationally representative data on the access to EID services and retention in the EID testing cascade. Infants who are started on treatment early in life are known to control viral replication better. Since early diagnosis is so crucial in program settings, there is a need to measure the timeliness of sample collection, test results, and completion of testing cascade and to identify reasons for delay along the testing cascade. Therefore, this study was conducted to assess the access to HIV testing, timeliness of EID services and retention within the testing cascades and the underlying determinants of the same.

## Method

### Study design

The study employed a mixed-methods design where the quantitative component was a secondary analysis of EID testing data reported by selected states and data maintained at sampled ICTCs. The analysis of data was aimed to quantify the gaps pertaining to proportion of HIV exposed infants with access to HIV testing and the attrition from the EID services. The qualitative component aimed at understanding the factors for the identified gaps with regard to demand and supply side challenges in the EID services. Qualitative data collection included in-depth interviews of various stakeholders at the state, district and ICTC level including caregivers of the HIV exposed infants. Use of mixed-method design helped in quantifying the EID Programme gaps and understanding the reasons for these gaps.

### Sample size estimation

The key variable was the percentage of HEIs with access to early diagnosis of HIV. The NACP provides the EID service at 6 weeks of age. We considered 8 weeks as the time appropriate for collection of first sample for early infant diagnosis consistent with the literature. We assume that 50% HEIs would have access to EID; i.e., first sample for DNA PCR is collected from 50% of HEIs by eight weeks of age. The estimated sample size with 5% absolute precision and 95% confidence interval and design effect of 1.5 (multistage sampling) was 576 HIV exposed infants. Number of pregnant women with HIV was the starting point and considering 25% loss due to pregnancy wastage, the final estimated sample size was 720 HIV positive pregnant women.

### Sampling strategy

**Figure 1:**
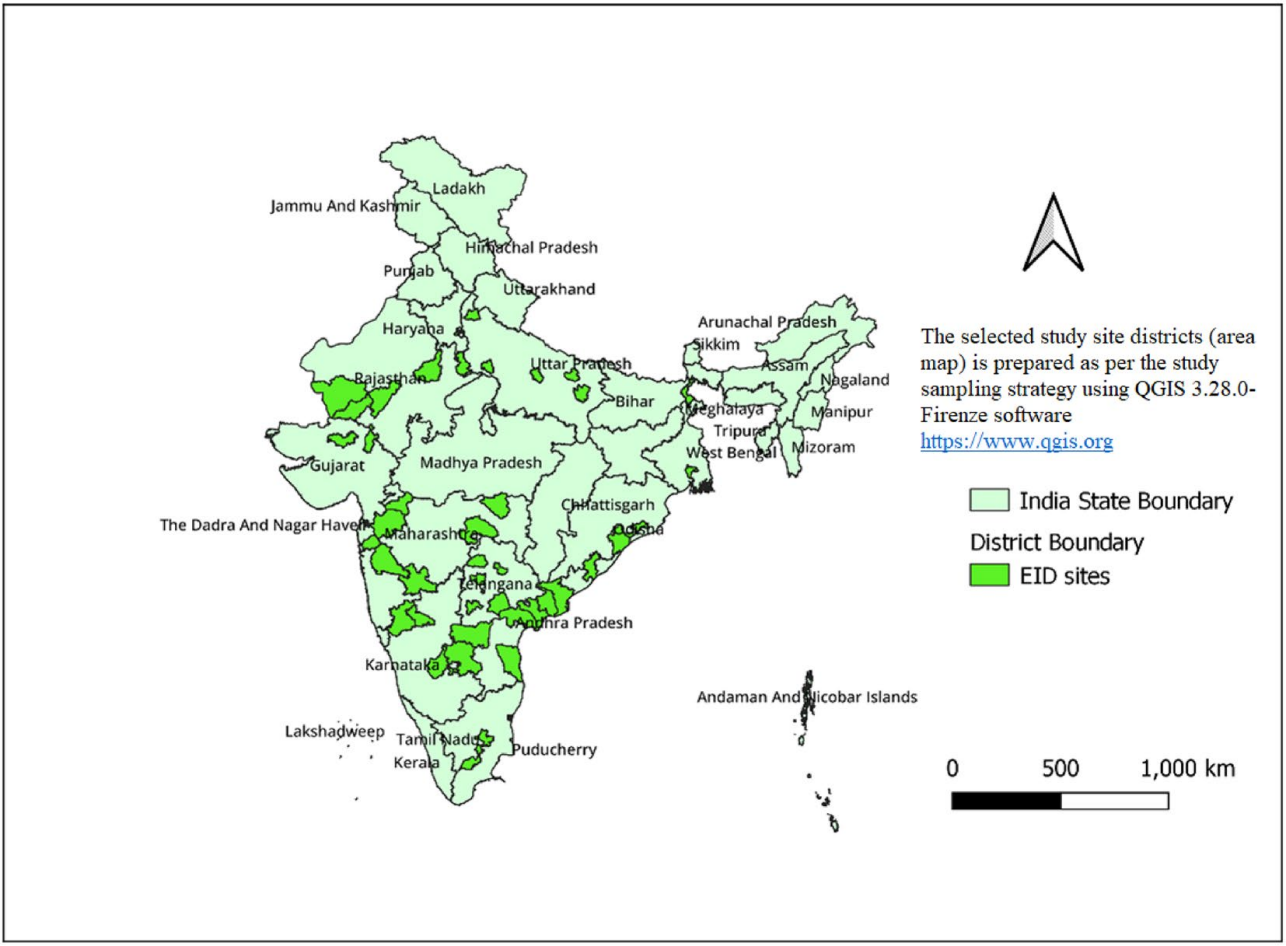
Geographic distribution of the selected ICTCs (districts shown in map) for assessment of EID program, India. (*Note*: The Fig. 1 to illustrate the selected study site districts (area map) is prepared as per the study sampling strategy using QGIS 3.28.0-Firenze software, QGIS Geographic Information System, QGIS Development Team (2021), Open Source Geospatial Foundation Project, https://www.qgis.org by the study team. Any use or reproduction of this figure requires approval from corresponding author).

A total of 11 states accounting for 80% of HIV positive pregnant women in India were included in the sampling frame. Reports from all ICTCs (3737) from these 11 states for reference period April 2015 to March 2017 were collected. The number of ICTCs that diagnosed more than 10 HIV positive pregnant women during the reference period were operationally defined as high case-load ICTCs, those with 1–10 as low case-load ICTCs. There were 331 high-case load and 2192 low case-load ICTCs. The rest 1214 ICTCs had no HIV positive pregnant women. In order to achieve the sample size of 720, a total of 50 high case-load ICTCs were sampled from among the 331 ICTCs using proportional to population size (PPS) sampling method. Only one of these 50 was not an EID-ICTC but was linked to the nearest EID-ICTC and data was collected also from this EID-ICTC. In order to get insights from low case-load ICTCs, one low case-load ICTC was selected per state. Overall, 62 ICTCs were selected (9 each from Andhra Pradesh and Maharashtra, 7 from Telangana, 6 each from Karnataka, Rajasthan, and Uttar Pradesh, 5 from Gujarat, 4 each from West Bengal and Delhi and 3 each from Odisha and Tamil Nadu) (Fig. 1).

### Quantitative tools

A secondary data tool was designed using the existing fields in paper-based records maintained at the ICTC. A list of HIV positive pregnant women was obtained from the PPTCT (Prevention of Parent to Child Transmission) beneficiary register or line list of HIV positive pregnant women or counselling register for pregnant women for the reference period (April 15 to March 16). Data on sample collection from HIV exposed infants was collected from the Exposed Infant/ Child register (EIC) and/or HIV-1 DNA PCR Test Requisition cum Results Form (TRRF). Data pertaining to particulars of mother and baby pair, receipt of PPTCT services, type of test (DNA PCR test or antibody test), time of collection and dispatch of samples for HIV testing, the results including the date of testing were extracted from the records and entered in a hand-held device.

### Outcome variables and analysis

Outcome variables included access to HIV testing services, timeliness of early infant diagnosis, and retention in EID program. The access to HIV testing services was defined as the proportion of HIV exposed infants who had at least one HIV test. Timeliness of early infant diagnosis was the proportion of infants with sample collection within 8 weeks of birth. Retention in EID program was defined as the follow-up of the infants up to 18 months of age or till confirmation of HIV diagnosis whichever was earlier. Explanatory variables for retention in EID services included mother’s education, parity, sex of the HEI, adequacy of ART to mother during antenatal period (24 weeks or more), cost of travel to EID-ICTC and whether the infant’s sample could be taken at the same or different facility of mother’s registration. Multivariate logistic regression analyses were performed to calculate adjusted odds ratios. Analysis was done using IBM SPSS 24.0.

Median times between a reactive antibody test and collection of a DBS sample for DNA PCR test, between sample collection and dispatch for DNA PCR and that between sample collection and testing for DNA PCR test were also calculated. The median time between a DNA PCR which detected HIV-1 and collection of the subsequent sample and between a sample without a conclusive report and collection of the subsequent sample were also calculated.

### Qualitative component

Semi-structured in-depth interview (IDI) guides were prepared separately for service providers, caregivers and state/district level program managers to understand their perspective on reach, effectiveness, challenges to EID services. Service providers included medical officers, counsellors and laboratory technicians of EID-ICTCs. The interview transcripts were translated to English and read and re-read. The transcripts were imported to Atlas-Ti V6.0. The quotes that explain the challenges to the utilization of EID services were noted. A code book was prepared and codes were assigned to the selected quotes using Atlas-Ti. Thematic analysis was carried out to help identify themes underlying challenges to EID services both from the provider and caregiver perspective. Quantitative and qualitative data findings were integrated in the “Result” section and further discussed in the discussion part to better understand the accessibility, timeliness and retention in EID services and the underlying factors that enable or hinder achieving these programme outcomes.

### Ethics approval and consent to participate

The study was approved by the Institutional Ethics Committee of ICMR National AIDS Research Institute, Pune (Ref no: NARI/EC/15-16/145), Institutional Review Board of Tata Institute of Social Sciences, Mumbai (Ref no: TISS/IRB/19Dec2014), NACO ethics committee (Ref no: 02/14/TRG). The written Informed consent translated in local languages were recorded from all the participants (health care providers, program managers and caregivers). No participants were under 18 years old. All research study methods were carried out in accordance with relevant guidelines and regulations.

## Results

Results are described under five broad sections.The quantitative data was analysed to estimate the gaps for accessing HIV testing and its timelines (section 1), retention proportion in HIV testing cascade (section 3) and factors associated with the EID testing (section 5).While qualitative analysis was used to understand the underlying factors for these gaps and described in section 2 (factors influencing access to and timeliness of EID services) and section 4 (factors for retention of follow up HIV testing).

### Section 1: Access to HIV testing services

In the period April 2015–March 2017, EID services were available at 1602(42.9%) facilities (EID-ICTCs) out of the 3737 SA-ICTCs. Out of the 13,321 HIV positive pregnant women diagnosed during the evaluation period, 10,662(80%) were diagnosed at the EID centres whereas the rest 20% didn’t have access to EID services at their ICTCs. The latter were expected to visit another SA-ICTC (EID centre).

**Figure 2:**
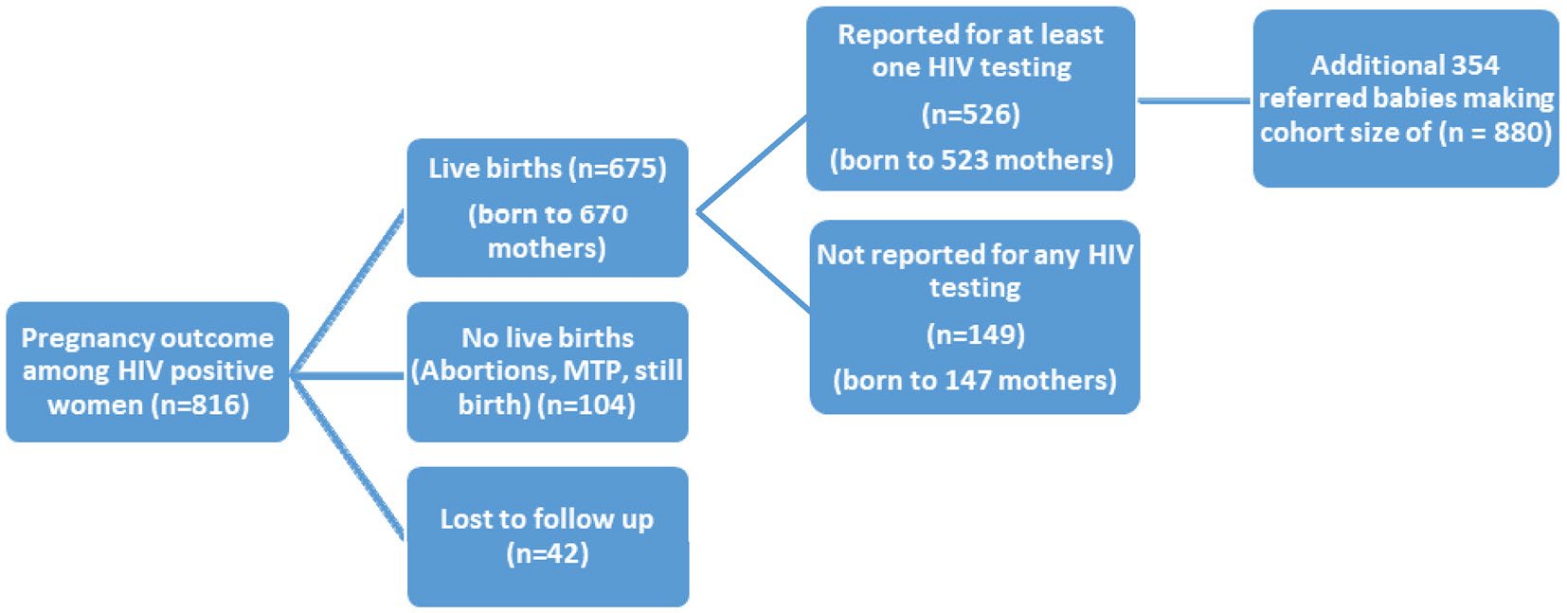
Pregnancy Outcomes and Access to HIV testing among infants born to mothers with HIV at the selected ICTCs (2016–2017) (n = 816).

The total number of pregnant women diagnosed with HIV at the 62 selected ICTCs was 816. A total of 670(82.1%) women had live births including twins resulting in a total of 675 live births (Fig. 2). Out of the rest, 42(5.1%) women were lost to follow up where as 104(12.7%) had abortions (12) or medical termination of pregnancy (53) or stillbirth (39). Out of the 675 live-born, at least one HIV test was conducted for 526(77.9%) infants (born to 523 mothers). The remaining 149(22.1%) infants (born to 147 mothers) did not have any HIV tests as per records at the selected ICTCs.

### Timeliness in HIV testing

**Figure 3:**
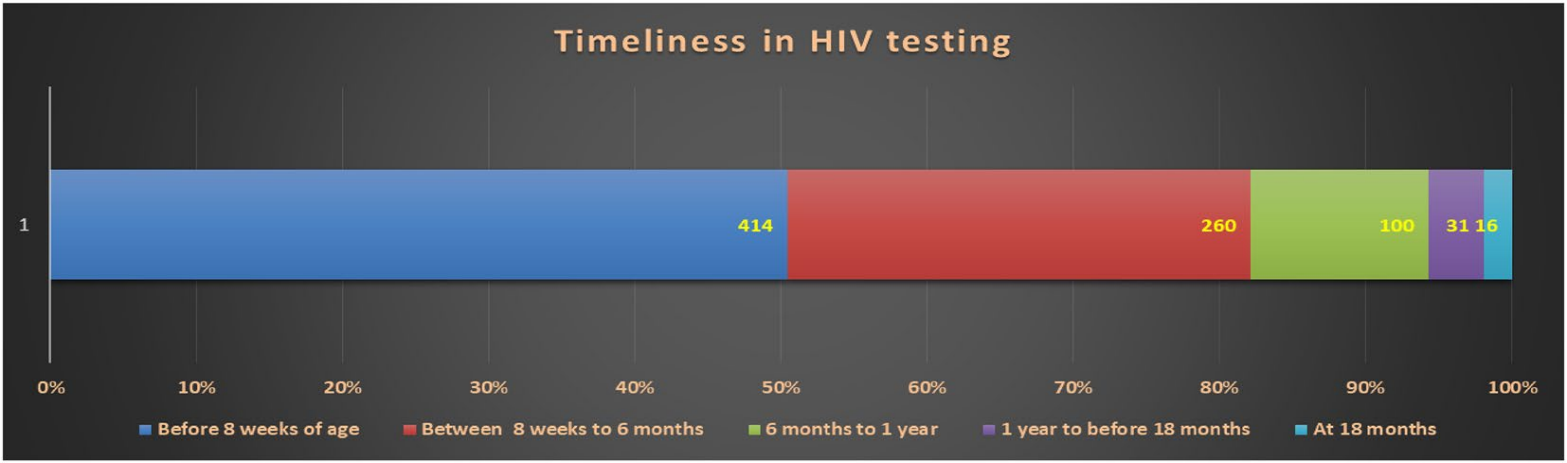
Timeliness of first sample for HIV testing among HIV exposed infants (n = 821).

The sampled ICTCs also received 354 infants during the reference period who were born to HIV positive pregnant women diagnosed at other ICTCs which lacked EID services. Thus, a total of 880(526 plus 354) infants underwent HIV tests. Of the 880, age details were available for 821 infants; 414(50.4%) of whom had their first samples drawn before 8 weeks of age fitting to the operational definition of ‘Early Infant diagnosis’ (Fig. 3). A total of 260(31.7%) had first samples drawn between 8 weeks to first six months of life, 100(12.2%) more during 6 to 12 months and 31(3.8%) had first test after their first birthday but before 18 months and the final 16(1.9%) got the 18-month antibody test as their first HIV test.

### Section 2: Demand and supply side factors influencing access to and timeliness of EID services

#### 2a. Supply

As stated already, EID services were made available at select facilities and that remains an important supply side factor. Availability of DBS cards at EID facility was another limiting factor. Isolated cases were reported by counsellors where ICTCs did not have DBS cards and had to ask mother to visit again.

The providers and state officials voiced the significance of counselling and counsellors as important resource on supply side. Counsellors were the first contact point who informed caregivers about the baby’s EID tests. The counselling process helped motivate caregivers to access EID services for the HIV exposed infants. *“If the counsellor is good enough that depends upon the rapport that develops between the beneficiary and service provider. If that is good enough, she (mother) will automatically come up.”—*a SACS official.

#### 2b. Demand

Stigma and discrimination, limited mobility/autonomy of women, marital relationship, family support/infringements, livelihood responsibilities, lack of felt need were common reasons for poor utilisation expressed by both healthcare providers and the women themselves.

*Stigma and discrimination* Mothers were the primary caregivers for the HIV exposed infants and usually they brought the babies for testing to the SA-ICTCs. Being a woman with HIV, they faced considerable stigma and discrimination. They were apprehensive of their HIV status getting disclosed in the community which limited their access to EID services.

*“…If they are daily labourers, they will not get work if people get to know. They face problems to live in neighbourhood. They can also lose their work. So, because of all these things, they don’t want to come to centre for services.”*—an ICTC counsellor.

*“I am poor, so I came here. I don’t have any problem in testing here. But I have fear that some people may talk bad if they came to know about this.”*—a caregiver.

*Family support:* The mothers voiced the lack of support from family (in-laws) although a few did report supportive environment. At the family level, stigma, discrimination due to HIV did play a role and so did the patriarchal norms that determine the autonomy and/or mobility of women.

*“They asked us where we were going. I told them that we were going to take medicines that my leg was paining. Today also, my father-in-law asked me where I was going, I told that I have pain in my legs so I am going to take medicines. By God’s grace, if my child’s report comes normal then we can hide. But if child is not negative, then they will get to know where we were going.”*—a mother.

*“Due to family problems, like their in-laws are against sending them for treatment, saying ‘you have this infection, then what’s the point in getting your child treated, anyhow it is not going to survive’. Sometimes, even husbands are against sending them for treatment. In this way, because of familial problems, babies are availing delayed services. In some cases, maternal relatives also refuse treatment for baby, and even if we approach their homes for convincing, they bluntly refuse it.”*—an ICTC counsellor.

*Mobility* Mobility of women was limited especially after delivery. They were not allowed to go out after delivery for 2–3 months. Husbands were usual escorts and women’s visits to hospital depended on the availability of husband to accompany. Mothers couldn’t reveal their HIV status to other family members.

*“Many times, husband stays outside. If they come with someone else, then they will get to know. Couples keep things only between them. If they have to tell other person, then there are chances of leaking that information. So, when her husband comes, then only she comes with her child.”*—an ICTC counsellor.

*Marital relationship and support from natal kin:* Women’s first source of support was husband; less did they expect from in-laws. However, the support from husband was not guaranteed especially in case of discordance. Divorce and destitution were not uncommon, natal home was the only hope. Absence of support posed challenges to accessing health services.

*“…sometimes due to circumstances of family, due to mother and father’s personal problems, they leave each other. Sometimes mother want to come for EID but father don’t want to take for EID. When we call them, they do not receive the call and we contact ASHA worker to contact patient, then they tell ASHA worker to tell us that they are out of station.”*—an ICTC counsellor.

The task of counselling HIV discordant couples was challenging even for counsellors with experience in the system. *“The acceptance of the female client becoming positive and the husband is negative is really difficult.”*—a State Official.

When mothers were left or divorced, they stayed with their parents. In such cases, all financial responsibility was borne by parents that put extra burden on families. Mother reported*: “Nowadays I am living with my parents and they took all financial responsibility of my delivery and I come here to take medicine every month and my parents paying ticket for that.”*

*Ignorance* Some felt that they did not have the disease nor do their child has. In such cases, the caregivers refused to undergo tests. Counsellors took help from NGOs to intervene in such cases and tried to link them to services. Due to this lack of felt need among the caregivers, some infants were never brought to ICTC for sample collection.

*“Patients are coming from remote areas. They will not know about HIV. They will say “I am healthy. Why are you saying that I am having this disease? Why I have to test my child?” Even if we say that they are HIV positive they cannot understand. Due to this ignorance, they will think that why we are calling them often unnecessarily.”*—An ICTC counsellor.

### Section 3: Retention in HIV testing cascades

**Figure 4:**
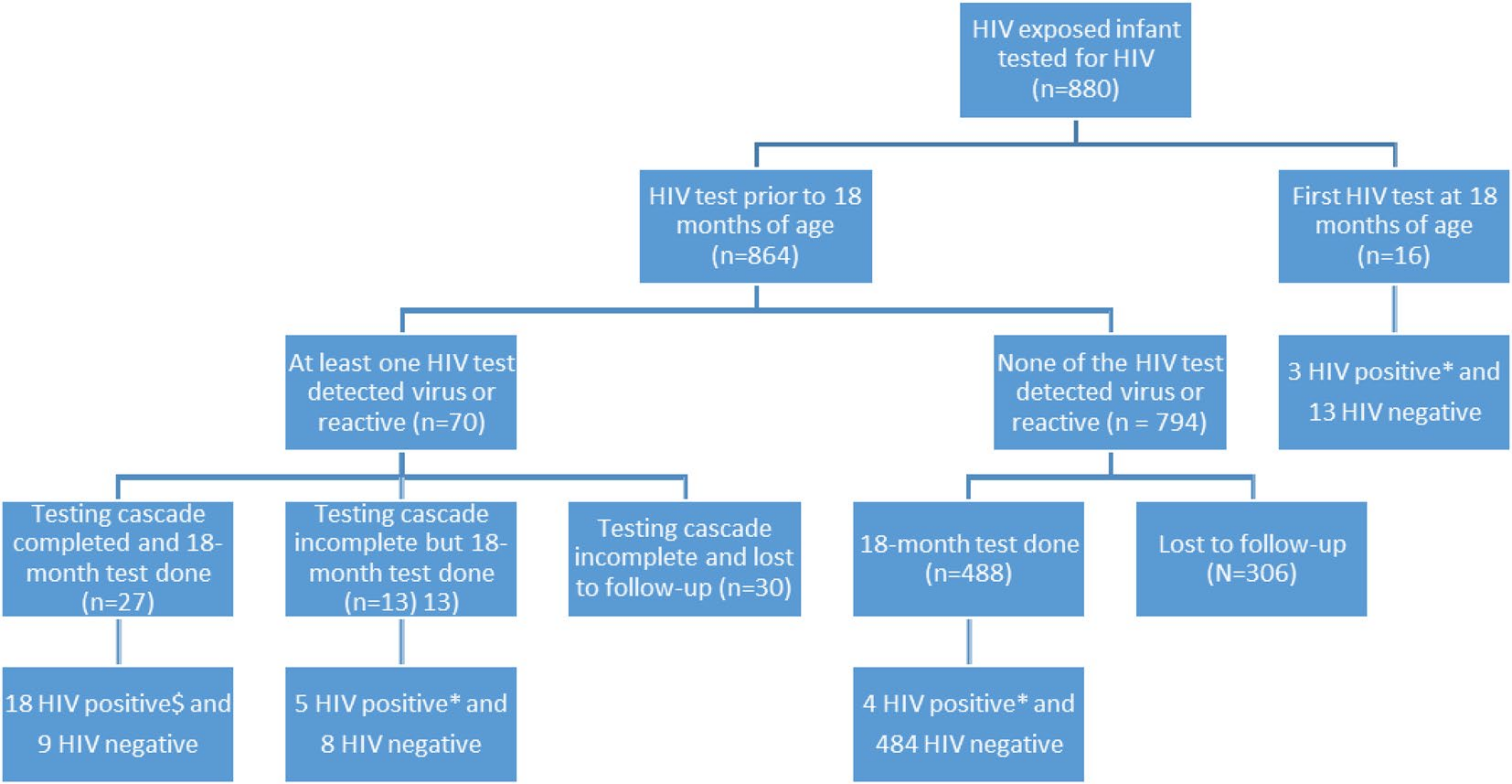
Flow-chart of retention in EID testing cascade among HIV exposed infants with at least one HIV test (n = 880). $ HIV diagnosis confirmed by detection of HIV-1 in two DNA PCR samples. *HIV diagnosis confirmed by 18-month antibody test.

Out of the 880 infants who underwent a HIV test, 16 tested only at 18 months. Of the rest 864, DNA PCR test detected HIV-1 in 40 infants. In another 19 infants, antibody test was reactive after a negative DNA PCR whereas 11 infants presented first time only after six months of age and had their antibody test reactive for HIV. Thus, a total of 70 infants had at least one HIV reactive test during the first 18 months whereas 794 did not have a single reactive test (Fig. 4).

Out of the 70 infants with a HIV reactive test, the cascade of two (or three) DNA PCR tests was completed for 27 of them and 18 were diagnosed with two DNA PCR tests detecting HIV-1 virus. The median age at diagnosis was 155 days (IQR 122.5–274.25 days; n = 17). Among the rest nine, both the DNA PCR tests subsequent to the reactive test did not detect the virus and the HIV negative status was confirmed by an 18-month antibody test.

For the rest 43 (out of the 70) infants, the required number of subsequent tests in the cascade were not conducted; 10 not getting first, 25 not getting second and three did not get the tie-breaker third DNA PCR test and five did not get the 18-month antibody test. However, 13 of these 43 infants received the18-month antibody test which helped determine their HIV status (five diagnosed with HIV).

Out of the 794 infants who did have at least one HIV test but none reactive during first 18 months, 488 had 18-month antibody test and four were diagnosed with HIV. Three of these four actually had only one HIV test during the first 18 months. The rest 306 were lost to follow-up and their HIV status could not be determined.

Thus, a total of 544(61.8%) were retained till 18 months of age in the testing cascade. A total of 30 (5.5%) were detected HIV positive (18 with two DNA PCR reactive tests and 12 with the 18-month antibody test). Pooled data of all these 30 infants had a median age of 421 days (IQR 149–650) at time of diagnosis of HIV.

### Section 4: Demand and supply side challenges affecting retention in HIV testing cascade and delays

In addition to the already presented demand and supply challenges, continuing in the testing cascade had a set of challenges.

#### 4a. Supply

Availability of EID-ICTCs, turn-around-time and human resources to follow-up on the HIV exposed infants were supply side factors.

*Availability of EID-ICTC* EID services were limited to a few facilities affecting the follow-up.

*“Sometimes people who come from other districts also visit this EID centre, so the proper follow up of this case from different villages and different districts is not done.”*—a DAPCU official.

*Turn-around-times* A major reason for the incomplete cascade was the turn-around-time for the test. The delay in getting the results for the first sample collected acted as a huge disincentive for follow-up tests. Counsellors faced challenges in explaining to the caregivers in cases where the results had not been received in time.

The median time to send the samples from the date of collection was five days (Table 1). Counsellor or Laboratory Technicians had to make arrangements for the transport and at times used their own money for courier which was not reimbursed in time. If ICTC personnel had to travel, then samples were transported periodically but not daily resulting in delay in transport. The median time from collection to testing was 36 days. Most often the DNA PCR samples were collected same/next day if the antibody test was reactive prior to 18-month but in almost a quarter of infants this time gap exceeded three weeks due to DBS cards’ unavailability.

**Table 1:** Median time for steps in early infant diagnosis cascade.

Early infant diagnosis cascade step	N	Median days (IQR)
Time between collection of DNA PCR and sending to regional reference laboratory	487	5 (2–11)
Time between collection of DNA PCR and testing at regional reference laboratory	407	36 (19–70)
Time between antibody test reactive and collection of DNA PCR	20	1 (0–23)
Time between collection of subsequent DNA PCR after a DNA PCR detected HIV	24	66 (54.5–116.25)
Time between collection of subsequent DNA PCR after a DNA PCR was indeterminate or sample was rejected	21	135 (98–161)

Time between HIV-1 detection at RRL and the infant’s subsequent sample being collected to confirm diagnosis was 66 days. After the testing, the results had to be conveyed to ICTC counsellor and then to the caregiver. The caregiver had to be then pursued to visit the ICTC at the earliest. There had been cases where the caregivers would visit EID centres many times but did not receive reports, and therefore they did not want subsequent tests.

*“They collected sample from the heel, there was a small paper, and they collected blood like this. After that, results are not given. I have called them so many times, but still results have not come. They said that—when result will come, we will let you know.”*—a caregiver.

*“We are not giving those results in 3 months or 6 months or 8 months and then we talk about regular follow up. We can give them date once or twice, but after that we have to tell them like a government system, we will let you know when your report comes”*—an ICTC counsellor.

*Human resources* The counsellors reported attempting home visits to motivate the caregivers for completing the EID testing cascade but not all could be reached (migration, long distances, stigma and discrimination, etc.). Outreach workers in the PPTCT interventions used to track patients earlier but with curtailing of older interventions, the follow-up became challenging.

*“Before we had outreach workers for babies follow up (PPTCT plus), a team in every hospital. Team means five members, if any ANC has come positive today, that ANC who is a client will be visited to their home by outreach workers, then they do house verification, every month they use to follow up with them, after delivery till 18-month, they used to give reminders for tests. Now, they are not here, staff is not there, because of that, the gap is increased, patients are coming late, or directly coming at 18 months. It has to be improved, if outreach staff is available, then it is better.”*—a SACS official.

#### 4b. Demand

Complex algorithm of EID, time and cost for repeated tests were key challenges.

*Complex algorithm* The EID testing algorithms included collection of multiple samples. Reactive antibody test and DNA PCR detection in first sample are not enough for diagnosis. Counsellors reported that explaining these becomes a daunting task. The need for repeated samples was not easy to comprehend for the caregivers. This meant delay and/or refusals.

*“6 weeks is ok, 6 weeks they come. Once they find out its negative, so for 6th month follow up they will be reluctant. Once they have come to know that it’s negative, for 6 months they will say, anyways it is going to be negative, so I don’t want to get my child tested again, pricked again and all that.”*—a ICTC MO.

*“At 6 weeks, we do a test and it will be negative and again after 6 months, the patient will be positive. Some of the cases refuse to believe that the child is infected.”*—DAPCU official.

Often the caregivers didn’t remember how many tests were done and when was the test done. They relied heavily on phone calls by the counsellor and then came for subsequent tests.

*“I don’t know about that, whenever blood test is required, they call us. When report comes, they call us.”*—a caregiver.

*Time, cost, and other pressing needs* Completing testing cascades meant repeated visits to the EID centres. Time, cost and livelihood posed challenges to follow-up visits. Mothers would be accompanied by male relatives which posed issues of losing daily wages.

*“They are labourers, farmers and daily labourers and they did not have money to come. And they have no time to come.”*—a ICTC MO.

*“We come by bus; it takes us one hour to reach here. We had to spend 250 INR. for the travel. And it takes whole day. We start in the morning and all work is done by evening.”*—a caregiver.

### Section 5: Factors associated with EID testing

Table 2 shows that access to HIV testing was significantly better among those infants whose mothers had adequate duration of ART (more than 24 weeks) (OR 2.89; 95% CI 1.35–6.17). Timeliness of the first sample collection was significantly lower (OR 0.31; 95% CI 0.10–0.92) among those infants whose samples were collected at centres different from those where their mothers were registered. In the unadjusted analysis for retention in testing cascade at 18 months was significantly higher among the mothers with secondary education than those less educated (OR 2.04; 95% CI 1.27–3.28), those who had ART for at least 24 weeks (OR 1.88; 95% CI 1.14–3.11), and those who had EID services at the facilities of their registration (OR 1.96; 95% CI 1.49–2.59) (not shown in the table). Mean travel cost for those who were retained was INR 104 per person statistically higher than that for those who could not be retained was INR 143. The adjusted analysis showed that receipt of ART for more than 24 weeks remained a statistically significant factor.

**Table 2:** Determinants of retention in early infant diagnosis (EID) testing cascade among HIV exposed infants.

Variables	Access to HIV testing	Timeliness of first sample collection	Retention in testing cascade till 18 months of age
	Adjusted odds ratio (Confidence interval)		
Education of mother			
Primary or less	Reference		
Secondary or more	1.04(0.57–1.89)	1.48(0.90–2.45)	1.50(0.83–2.71)
Parity			
-First	Reference		
-Second or higher	1.06(0.59–1.90)	0.80(0.49–1.32)	0.98(0.55–1.74)
Duration of Anti-retroviral therapy received by mother			
Less than 24 weeks	Reference		
24 weeks or more	2.89(1.35–6.17)*	1.49(0.89–2.50)	1.96(1.03–3.71)*
Sex of HIV exposed infant			
Male	Reference		
Female	0.81(0.45–1.44)	1.28(0.78–2.12)	1.08(0.60–1.94)
Facility where infant’s sample was collected	Not applicable		
Same where mother is registered		Reference	
Different from that of mother’s registration		0.31(0.10–0.92)*	6.08(0.78–42.19)
Cost of travel to EID centre		1.00(0.99–1.00)	1.00(1.00–1.01)

*p < 0.0

## Discussion

The paper highlights the barriers to access, timeliness and retention in HIV testing cascade in India’s EID services. This is the first such programme evaluation of national wide EID services to find out the reasons underlying the lack of access, delays and loss to follow-up. The study highlights that access to EID services is not still universal across healthcare facilities and that the access to HIV testing was 78% among HIV exposed infants. Only half of the infants who are receiving the test are receiving it within 8 weeks i.e., ‘early’ diagnosis and more than a third are lost to follow-up.

Diagnosis of perinatally acquired HIV infection is contingent upon diagnosis among pregnant women. Nearly 20% of Indian women did not have access to HIV testing in 2019–2020^2^. At the national level, the NACP is able to detect about 15,000 HIV positive pregnant women out of the estimated 22,000. This is a gap in the prevention of parent to child transmission of HIV (PPTCT) services which limits diagnosis among infants. The testing among pregnant women needs to be made universal. The role of the National Health Mission (NHM) is crucial in provision of maternal healthcare. To improve coverage and testing, both NACP and NHM would need to build greater synergies amongst each other. Further research is needed to study synergies between the National Health Mission (NHM) and the National AIDS Control Program (NACP) in India.

This study found that even for the HIV positive pregnant women known to the programme, one in every five infants could not access HIV testing during the study period. South Africa has piloted interventions with testing at birth a novel model with testing both at birth and 6 weeks has been found to be cost-effective^9^ but the follow-up became weaker^10^. In India, one of every five deliveries occurs at home^11^. It will be logistically challenging to make EID services available at every facility with delivery care due to wide geographic area of the country. Decentralisation of existing services may be useful and discussed below.

The EID involves cascades where repeated samples are needed and they need to be transported to far-off facilities. There is a need to decentralise the EID testing. Under the programme, HIV viral load testing laboratories are being established across the country and major states have viral load testing infrastructure. Such a partial decentralisation of EID testing to state-level will reduce the Turn-around-time (TAT); however, a very low testing load at the state-level would make this operationally infeasible. Our study found that those mother-baby pairs who did not have the facility of EID sample collection at facility of mother’s registration had delays in the sample collection. Most of the literature on EID is from sub-Saharan Africa where decentralisation to local levels has been found to be successful^12^. However, in Indian settings the prevalence is low even among relatively high-burden states when compared to the sub-Saharan African settings. In the study, there were most facilities had less than 10 HIV positive pregnant women in two-year period. Assuming these infants undergo all three tests (at birth, at 6 and 12 months of age), the total number of EID sample collections would be 3–27 in the two-year period; which means hardly one or two infants at a facility in a month. With such a low volume of service delivery, the decentralising sample collection at facilities will be logistically challenging and it will still have challenge of the turn-around-times with testing facility located at a far-off distance. Decentralisation to most periphery warrants point-of-care tests. The accuracy of the nucleic acid point-of-care (POC) tests is high^13^ but the context of the country and existing infrastructure is an important consideration^14^. The number of HIV positive pregnant women per ICTC is low in the low prevalence Indian setting. Providing the services at the vast number of facilities with very little annual load may pose a challenge from a logistics, quality and management point of view. A distinct possibility is to offer it at ART centres where mothers are expected to visit for their own treatment and care. The acceptability, feasibility and effectiveness of this decentralised point of care test is an area for implementation research.

In the Indian programme, communication of test results from reference laboratories to ICTCs shifted from paper-based couriers to emails/mobile messages. Although we could not quantify the time between testing date and date of receipt of results at ICTC, a systematic review found that about 2.5 weeks can be saved by using SMS/GPRS technologies compared to conventional courier of paper-based reports^15^. The communication of test result from the ICTC to the caregiver remains a challenge. In our study median time from sample collection to testing was 36 days but time to next sample collection (in case of a positive result) was 66 days. This indicates that nearly one month is lost between the test result being positive and the infant being brought back for the next sample. Kenyan program had a comprehensive messaging system which apart from delivering the message to caregivers about availability of results also alerts them when the infant is due for a test. This algorithm is complex. It is possible that counsellors may also get confused about which test to perform and when. An application based upon a database can be the best solution to send reminders to the counsellors to follow up. The Kenyan system also uses algorithm-driven electronic alerts to providers and laboratory technicians as well. The effectiveness has been demonstrated by a cluster randomized trial^16^. In this study, 12 out of the 30 confirmed diagnosed cases were at the age of 18 months in spite of the presence of EID services. In order to improve the delay, there is a need for testing interventions that would make use of technology to remind caregivers as well as counsellors to improve the timeliness.

The communication to caregivers may still not be able to result in the infants being brought to the sample collection facility due to several barriers. Stigma and fear of discrimination, destitution are prominent reasons in this study and have been well documented in previous literature^17^. Accompanied referral^18^, peer and lay person support^19^ and involvement of male partner^20^ have been attempted in African nations with success. In Indian settings, one needs to explore the potential of positive people network to facilitate infants being brought to the peripheral facility for sample collection. NACP has engaged with NGO partners to support Elimination of Mother to Child Transmission (EMTCT). These workers and those in the healthcare delivery system such as ANM and ASHA may support the referral of the caregivers to the EID-ICTCs. This also is an area of implementation research but it should also take into consideration the privacy and confidentiality while reaching out to the caregivers at their homes. This is important because of the persistent stigma around HIV epidemic. The stigma in low-prevalence setting of India and the means to address it during implementation research would be very different from high-prevalence settings of Africa where most research has taken place^17^. This would be of prime importance not only for designing such an implementation research but also for broader stigma reduction interventions at the societal level grounded in the Indian settings where meagre research has taken place on stigma, its association with access to healthcare and effectiveness of stigma reduction interventions in the Indian context^21^.

As a result of the challenges, the retention in EID testing cascades till 18-months of age was possible only for 61.8% of those who entered the cascade. A recent review found that under program conditions only 57% of the HIV exposed infants are retained till 18-months either due to early death or lost to follow up^3^. Retention in the EID testing cascade was associated with education, travel cost, and receipt of ART for more than 24 weeks by mother in the present study. The findings are similar to other LMIC (Low-Middle-Income Countries) settings^22–24^. Mothers who are able to take ART are the ones with lesser barriers and also those who could be provided with counselling and education regarding early infant testing. Improving access to pregnant women to ART is therefore important both from prevention of transmission and ensuring follow-up of the HIV exposed infant. Travel reimbursements and direct benefit transfer are possible ways to facilitate the access by improving financial access.

The study had certain limitations. The study used secondary data available in the records at the sampled ICTCs. Data on certain variables was missing in some of the records and hence the data presented has varying denominators. It is possible that some of the reports received from RRL were not entered into the registers. The sampled ICTCs do not necessarily represent the wide variation in the country as a whole. As the date of receipt of test result from RRL and date of result communication to caregiver were not available, we could not estimate time from testing to communication of result to caregiver. Though we found that 30 infants were confirmed to have HIV transmission, it may be an underestimated as some did not complete testing or not reported for the testing. Data on treatment outcome of children diagnosed under EID Programme has been collected and in process of analysis. However, the study had several advantages. The mixed-method approach yielded a comprehensive picture in terms of programme implementation gaps and the contextual factors that affect these gaps from the perspectives of those implementing the EID programme (supply-side) and those utilizing the EID services (demand-side). In addition to improvement in EID programme, early ART treatment to mothers, ART adherence among them and nevirapine prophylaxis to new born babies will lead to elimination of maternal to child transmission of HIV (EMTCT). In spite of HIV transmission risk, breast feeding should be continued among HEIs till six weeks in India considering the mortality risk among non-breastfed children due malnutrition, diarrhoea, and acute respiratory tract infection.

## Conclusions

This comprehensive nationwide study highlights the implementation gaps in access, timeliness and retention for HIV testing under the EID program in India. Although 78% of HIV-exposed children accessed the EID services, only half of them accessed HIV testing services on time and 62% of them retained in the EID testing cascade. Both demand and supply-side contextual factors play an important role in timely access and completion of the EID testing cascade. The study results underline the need for evidence-based implementation strategies, such as—synergistic coordination between national programs, decentralisation of existing EID-HIV services to ART centres, technology-enhanced interventions to improve timeliness, involvement of community health workers and community networks for linkages to care and regular follow-up, and financial protection to improve EID services utilization.

## Data Availability

The datasets generated and/or analysed during the EID study are not publicly available due data accessibility and confidentiality policies of National AIDS Control program, India but de-identified datasets are available from the corresponding author on reasonable request.

## Abbreviations

EID: Early infant diagnosis
ICTC: Integrated Counselling and Testing Centres
ART: Anti-retroviral therapy
CHER: Children with HIV Early Anti-Retroviral Therapy
NACPV: National AIDS Control Program
SA-ICTC: Stand-Alone Integrated Counselling and Testing Centres
DBS: Dried blood spots
DNA PCR: Deoxyribonucleic acid—polymerase chain reaction
HEI: HIV-exposed infant
RRL: Regional Reference Laboratory
LMICs: Low-Middle Income Countries
PPS: Proportional to population size
PPTCT: Prevention of Parent-to-Child Transmission
EIC: Exposed Infant/Child register
TRRF: Test Requisition cum Results Form
IDI: In-depth interview
NHM: National Health Mission
TAT: Turn-around-time
POC: Point-of-care
EMTCT: Elimination of Mother-to-Child Transmission
